# Genomic and phylogenetic analysis of hypervirulent Klebsiella pneumoniae ST23 in Ireland

**DOI:** 10.1099/mgen.0.001373

**Published:** 2025-03-19

**Authors:** Mark Maguire, Niall DeLappe, Christina Clarke, Alma Touhy, Ulrike Carlino-MacDonald, Alan Hutson, Martin Cormican, Wendy Brennan, Genevieve Devane, Dearbháile Morris, Simone C. Coughlan, Georgios Miliotis, Thomas A. Russo, Liam P. Burke

**Affiliations:** 1 Antimicrobial Resistance and Microbial Ecology Group, School of Medicine, University of Galway, Galway, Ireland; 2 Centre for One Health, Ryan Institute, University of Galway, Galway, Ireland; 3 Research Ireland Centre for Research Training in Genomics Data Science, University of Galway, Galway, Ireland; 4 Galway Reference Laboratory Service, University Hospital Galway, Galway, Ireland; 5 The Veterans Administration Western New York Healthcare System, Buffalo, New York, USA; 6 Department of Medicine, University at Buffalo-State University of New York, Buffalo, New York, USA; 7 Department of Biostatistics and Bioinformatics, Roswell Park Comprehensive Cancer Center, Buffalo, New York, USA; 8 Department of Microbiology and Immunology, University at Buffalo-State University of New York, Buffalo, New York, USA; 9 The Witebsky Center for Microbial Pathogenesis, University at Buffalo, State University of New York, Buffalo, New York, USA

**Keywords:** carbapenemase, hypervirulence, *Klebsiella pneumoniae*

## Abstract

Hypervirulent *Klebsiella pneumoniae* (hv*Kp*) has emerged as a pathogen of global concern associated with invasive community-acquired infections. The combination of hypervirulence and carbapenem resistance can result in severe and difficult-to-treat infections. This retrospective study aimed to investigate the spread of hv*Kp* sequence type 23 (ST23) in Ireland and the convergence of hypervirulent (hv) and antimicrobial resistance genotypes. Short-read sequences (PE300) for 90 *K. pneumoniae* ST23 isolates were generated by the Galway Reference Laboratory Services (GRLS). Isolates were from screening swabs (*n*=59), invasive infections (*n*=18), non-invasive sites (*n*=12) and the hospital environment (*n*=1). The virulence and resistance content were assessed genomically using Kleborate (v2.2.0), ABRicate (v1.0.1) and Platon (v1.6). The *in vivo* virulence of the isolates was assessed using a murine model. All isolates were genotypically hv with 88/90 isolates having a maximal Kleborate virulence score of 5 including carriage of key genes. Eighty-two per cent of isolates (74/90) carried a carbapenemase gene (*bla*_OXA-48_/*bla*_OXA-181_/*bla*_NDM-1_), and 42% carried resistance genes to 3 or more antimicrobial classes. Core genomic delineation revealed the isolates to be clonal with similar resistance and virulence profiles. Two distinct clusters of Irish isolates were detected consisting of 82/90 of the isolates. Isolates associated with carriage and infection demonstrated similar *in vivo* virulence. An established clone of hv*Kp* ST23 is circulating within Ireland and causing both colonization and infection of patients. The lack of reliable screening methods for hv*Kp* makes its detection and control in the healthcare setting challenging.

**Impact Statement**

This study provides insight into the spread of an established clone of carbapenemase-producing and frequently multidrug-resistant hypervirulent *Klebsiella pneumoniae* (hv*K*p) in Irish hospitals. We provide evidence supporting the *in vivo* hypervirulence of this clone, its capacity for persistent patient colonization and progression to infection. Our findings highlight the need for reliable methods to detect hv*Kp*, particularly in the healthcare setting. Enhanced surveillance will enable accurate detection and tracking, which is essential to prevent further spread of these highly virulent pathogens.

## Data Summary

All genomes are available at the National Center for Biotechnology (NCBI) under the project number PRJNA1078342.

## Introduction

*Klebsiella pneumoniae* is a common colonizer of the human gut and can cause a variety of infections. Two pathotypes designated as classical *K. pneumoniae* (c*Kp*) and hypervirulent *K. pneumoniae* (hv*Kp*) exist, and both are globally disseminated pathogens associated with severe infection. Whilst c*Kp* is most commonly seen as an opportunistic pathogen in the healthcare setting, hv*Kp* is usually associated with invasive community-acquired infections, often in healthy hosts [1]. Infections due to hv*Kp* have been associated with increased mortality and morbidity. Liver abscess is a common consequence of hv*Kp* infection, but infection of nearly every site in the body can occur, including endophthalmitis, necrotizing fasciitis, meningitis and brain and epidural abscess; multiple sites of infection are not uncommon [2].

Hv*Kp* was first reported in 1986 as the cause of a pyogenic liver abscess in Taiwan [3]. To date, infections caused by hv*Kp* have been mostly reported in countries in the Asian Pacific Rim [4,5] but are increasingly detected outside of Asia and particularly in Europe [6,7]. Hv*Kp* is associated with specific capsule types – the most common is K1 but K2, K5, K57 and others are also associated with hypervirulence [8].

Hv*Kp*-associated virulence plasmids (pVir, 140 to 220 kb) are the dominant genetic element responsible for the hv*Kp* hypervirulent (hv) pathotype [9]. The *rmpADC* operon located on pVir, which encodes for virulence factors that upregulate capsule production and confer the hypermucoid phenotype and the *iuc* operon which encodes for the siderophore aerobactin have been shown to contribute to the hv phenotype. *peg-344* is also present on pVir and has been demonstrated to contribute to pulmonary but not systemic infection [10]. pVir are not self-conjugative but are capable of mobilization by other conjugative plasmids, such as plasmids carrying antimicrobial resistance (AMR) genes [11,12]. Chromosomal acquisition of ICE*Kp*10, carrying *ybt/irb* (yersiniabactin siderophore biosynthesis) and *clb* (colibactin toxin biosynthesis), has been associated with the emergence and dissemination of the hv sublineage clonal group 23 (CG23)-I [9]. ICE*Kp* are self-transmissible but appear to be mainly capable of transmission within the *K. pneumoniae* species [13]. Other chromosomal genes present in both c*Kp* and hv*Kp* strains that encode for the siderophore enterobactin and genes linked to factors that contribute to biofilm formation (*mrk* and *fimH*) also contribute to the organism’s ability to cause infection and persist in a variety of environments [14,15] but are not specific to hv*Kp*.

Whilst c*Kp* is commonly associated with AMR, hv*Kp* strains have been largely susceptible to antimicrobials. However, there have been increased reports of resistant hv*Kp* strains initially stemming from Asian countries, but antibiotic-resistant strains have now been reported globally [16]. The acquisition by hv*Kp* of carbapenemase genes through highly mobile plasmids is of particular public health concern. This convergence of hypervirulence with AMR can result in strains capable of causing severe invasive infections with few treatment options and poor clinical outcomes [17].

*K. pneumoniae* ST23 is a globally recognized hv clone forming the predominant sequence type in the CG23 [18]. The first case of *K. pneumoniae* ST23 in Ireland was reported in 2019. It was detected in the context of an outbreak within hospitals and long-term care facilities in the South-Eastern region [19], and these cases prompted further investigation of hv*Kp*. This was based largely on a review of the sequence data from carbapenemase-producing *K. pneumoniae* submitted to the national reference laboratory service. This service has received and sequenced all first instances of carbapenemase-producing Enterobacterales (CPE) isolates detected in patients since 2018. This is linked to the national programme of testing hospital admissions for rectal colonization with CPE. In addition, isolates of *K. pneumoniae* suspected on clinical grounds as hv*Kp* from the outbreak-associated hospitals were submitted to the reference laboratory if not carbapenemase producers. The collection of isolates described here is therefore biassed towards carbapenemase producers. Carbapenemase-non-producing hv*Kp* associated with rectal colonization would not be detected and may represent a much higher proportion of hv*Kp* than is apparent from this study.

There is limited information on the virulence genes, AMR, genetic diversity, evolution and spread of hv*Kp* ST23 in Ireland. In order to address this, the present study sought to genomically characterize the hv*Kp* ST23 isolates collected in Ireland to date. We carried out *in silico* analyses to determine the resistome, virulome, plasmid content and phylogenetic relationship of these isolates. Furthermore, *in vivo* infection studies on a genomically diverse selection of isolates were performed to determine if the genotype correlated with virulence in a well-validated animal model.

## Methods

### Isolate selection

A total of 1769 *K. pneumoniae* isolates were referred to the Galway Reference Laboratory Services (GRLS) between 2019 and 2023, 1095 (62%) of which carried a carbapenemase-encoding gene (CEG). A total of 96 ST23 *K. pneumoniae* genomes were identified in the dataset. Of these, 90 passed quality control and were used in downstream analysis. The majority of these isolates carried a CEG (*n*=76, 84%); the genes detected were *bla*_OXA-48_ (*n*=73), *bla*_OXA-181_ (*n*=1) and *bla*_NDM-1_ (*n*=2). The isolates were associated with invasive infection (*n*=19 from normally sterile body sites), from rectal swabs or stool (*n*=59, colonization), from other anatomical sites (colonization or infection) (*n*=11) and an environmental site (*n*=1). These isolates were collected from 80 patients in 18 healthcare facilities (acute care facilities and long-term care facilities) across Ireland. To investigate within-patient persistence and progression from carriage to infection, ten serial isolates were included in the analysis where the samples were collected from the same patient but from different anatomical sites or from the same site but at least 1 year apart.

### Antimicrobial susceptibility testing

Minimum inhibitory concentration (MIC) testing for amikacin, aztreonam, ceftazidime/avibactam, ceftolozane/tazobactam, colistin, eravacycline, fosfomycin, imipenem, imipenem/relebactam, meropenem, meropenem/vaborbactam, piperacillin/tazobactam, tigecycline and tobramycin was carried out. The MIC was determined via broth microdilution using the Sensititre™ EUMDRXXF AST plate (Thermo Scientific™) and was controlled using *Escherichia coli* ATCC 25922 and *Pseudomonas aeruginosa* ATCC® 27853 in each batch. Results were interpreted according to EUCAST guidelines v14.0 [20]. Isolates were defined as multidrug resistant (MDR) if they were resistant to more than three different classes of antimicrobial agents tested.

### DNA extraction and whole-genome sequencing

DNA was extracted from isolates using the EZ1DNA Tissue kit (Qiagen GmbH, Germany) on the EZ1 Advanced XL instrument. Sequencing libraries were prepared using the Nextera DNA Flex/DNA Prep library prep kit (Illumina, CA), and whole genome sequencing was performed using the Illumina MiSeq platform to generate 2×300 bp paired-end reads. All isolates were sequenced as they were received in the GRLS between 2019 and 2023.

All genomes were assembled using Unicycler (v0.5.0) using default parameters at the start of this study to ensure uniformity and compatibility with downstream analysis. Genomes are available at the National Center for Biotechnology (NCBI) under the project number PRJNA1078342.

### Global *K. pneumoniae* ST23 genomic data collection

All available *K. pneumoniae* ST23-KL1 genomes (*n*=424) were downloaded from the Pathogenwatch database (https://pathogen.watch/ access date: 10 March 2024). Metadata and Kleborate output for these genomes were also obtained from the Pathogenwatch database. These genomes were predominantly of human origin (*n*=339) but also from animal sources (*n*=12), environmental sources (*n*=11) and food (*n*=1). The remainder of the genomes (*n*=61) were from unknown sources (see Table S6, available in the online Supplementary Material).

### Genotypic characterization of hv*Kp* ST23 genomes

Sequences were analysed using Kleborate (v2.3.0) [19,21] to identify the ICE*Kp*-associated virulence loci, virulence plasmid-associated loci and AMR determinants. Kaptive (v2.0.4) was used to identify capsule antigen (K-loci) and lipopolysaccharide antigen (O-loci) [21]. Genomes were also screened for the five virulence genes (*iucA*, *iroB*, *rmpA*, *rmpA2* and *peg-344*) that define a strain as hv*Kp* [22].

To analyse the plasmid content of the assembled sequences, Platon (v1.7) [23,26] was used to predict the plasmid-borne contigs and output them as a separate file. Platon was run in the default ‘accuracy’ mode. The files obtained from Platon were then analysed using ABRicate (v1.0.1) (https://github.com/tseemann/abricate) using the ResFinder [27], PlasmidFinder [24] and virulence factor [28] databases (downloaded 1 April 2024), using default settings of 80% identity and coverage, to determine the resistance genes, plasmid content and virulence genes, respectively. AMRFinder [29] was used to detect resistance-associated point mutations.

A core genome SNP alignment was generated with snippy (v4.6.0) (https://github.com/tseemann/snippy). Phylogenetic analysis was performed to infer the evolutionary relationships based on the core genome SNP alignment with RAxML-NG (v1.2.0) [30] using the general time reversible-gamma model with rapid bootstrapping of 100 bootstrapping replicates. The *K. pneumoniae* reference strain (GCA_000240185.2) was used as an outgroup for the phylogenomic inference. The phylogenetic tree was visualized using iTOL [31].

TreeCluster [32] was used to identify clusters of genomes from the tree using the default ‘Max Clade’ method and a threshold-free approach to maximize the number of non-singleton clusters (best threshold=0.00311).

### CD1 mouse subcutaneous challenge systemic infection model

To assess the *in vivo* virulence of selected Irish ST23 isolates, a murine infection model was used as described previously [33,34]. Animal studies were reviewed and approved by the University at Buffalo, State University of New York (SUNY), and the Veterans Administration Institutional Animal Care Committee. This study was done in strict accordance with the recommendations in the Guide for the Care and Use of Laboratory Animals endorsed by the National Institutes of Health [35], and all efforts were made to minimize suffering. In brief, outbred male CD1 mice (18 to 22 g; *n*=5 per group) were injected subcutaneously with various titres of the bacterial strains being assessed.

Eleven isolates were selected randomly from the different clusters identified and the different sampling sources and geographical regions within the collection. Six isolates from cluster 1 were chosen from invasive infections [blood (*n*=2) and liver abscess (*n*=1)], other infection [urine (*n*=1)], rectal screening (*n*=1) and hospital environment (*n*=1). Two isolates collected 2 years apart were selected from cluster 2. The remaining three isolates were selected from isolates that did not form a part of any cluster and were collected from healthcare facilities, which were not a part of either cluster.

Animals were monitored for up to 14 days for the development of the study endpoint, severe illness (in extremis state) or death, which was recorded as a dichotomous variable. The LD_50_ was estimated using a logistic regression model as described [22].

## Results

### Kleborate analysis of the Irish genomes

Genomes were identified as *K. pneumoniae* sequence type 23 (ST23) with 88/90 belonging to the capsule type 1 clade (ST23-KL1) and 2 genomes belonging to the capsule type 57 clade (ST23-KL57). Kleborate results demonstrated that all ST23-KL1 had a maximum virulence score of 5; i.e. the loci encoding aerobactin (*iuc*), yersiniabactin (*ybt*) and colibactin (*clb*) were all detected. The ST23-KL57 genomes had a virulence score of 4 (*iuc* and *ybt* loci only) (see Table S2).

### Characterization of hypervirulence-associated loci

The yersiniabactin locus and colibactin locus are highly associated with the ICE*Kp* present on the chromosome. All ST23-KL1 genomes carried the yersiniabactin locus *ybt1* located in ICE*Kp*10 (1/88 truncated). This variant of ICE*Kp* also carries the colibactin locus, which in 86/88 genomes was *clb2* (2/88 truncated). In the remaining genome, the *clb* locus detected was an incomplete *clb3*. Both ST23-KL57 genomes carried the *ybt9* variant located on ICE*Kp*3, which lacks the *clb* loci [13].

The virulence plasmid (repB_KLEB_VIR) was detected in all isolates and is associated with carriage of the salmochelin (*iroBCDN*) loci, aerobactin (*iucABCD* and *iutA*) loci, the *rmpADC* locus and *rmpA2* gene, which regulate the expression of the mucoid phenotype and *peg-344* [36]. The same salmochelin (*iro1*, 1/88 incomplete) and aerobactin (*iuc1*) loci were detected in all ST23-KL1 genomes, but the ST23-KL57 genomes lacked the salmochelin genes. The *rmpADC* locus was also detected in 89/90 (6/89 truncated) genomes, whilst a truncated *rmpA2* gene was detected in 79/90 genomes. The *peg-344* gene was also detected in all genomes. Eighty-six per cent (77/90) of isolates possessed all 5 of these biomarkers, which has been shown to be an accurate predictor of the hv*Kp* pathotype [9]; however, *rmpA2* was truncated in all.

The ST23-KL1 isolates carried a greater number of virulence genes overall (mean=127.3, sd=4.6) than the ST23-KL57 isolates (mean=85.5, sd=0.7). Other important virulence genes detected included the *entB* (siderophore), *mrk* (adhesin) and *fimH* (adhesin) genes, which were detected in all isolates (see Table S3).

### Characterization of AMR and plasmid markers

**Fig. 1.**
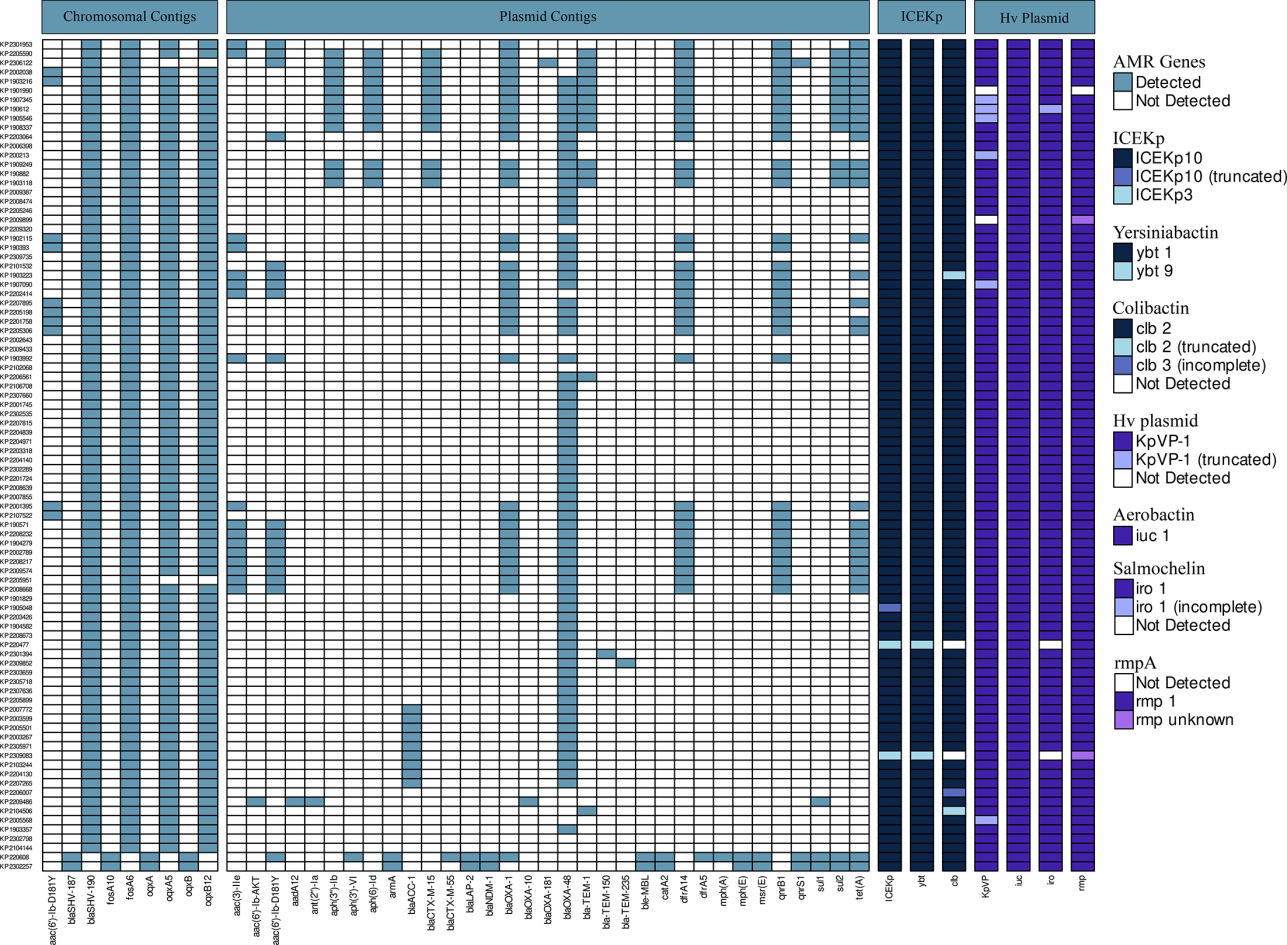
A presence–absence matrix of AMR genes and hypervirulence genes. Their predicted location on either the chromosome or plasmid is highlighted at the top of the diagram. The virulence genes are coloured according to the variant of the gene detected.

Analysis of the chromosomal contigs identified a highly conserved set of resistance genes on the chromosomes of the isolates within the ST23-KL1 cluster: quinolone efflux pumps *oqxA5* and *oqxB12* (86/88), fosfomycin resistance genes *fosA6* (88/88) and *β*-lactamase gene *bla*_SHV-190_ (88/88). The two ST23-KL57 isolates carried different variants of these genes: *oqxA*, *oqxB*, *fosA10* and *bla*_SHV-187_ (Fig. 1).

A single point mutation conferring resistance to carbapenems, ompK36(D135DGD), was detected in a single ST23-KL1 isolate (KP1901990). Three resistance-associated point mutations were detected in the ST23-KL57 isolates; these confer resistance to fluoroquinolones, gyrA(S83I) and parC(S80I), and carbapenems, ompK36(Q313STOP) (Table S5).

The total resistance gene content of the isolates varied from 4 to 21 resistance genes (mean=7.9, sd=3.8) with 73/88 ST23-KL1 genomes carrying a *bla*_OXA-48_ gene and a single isolate carrying a *bla*_OXA-181_ gene. The majority of these isolates (91.7%) were susceptible to the carbapenem antibiotics tested, in accordance with the low-level carbapenem resistance phenotype that is usually associated with *bla*_OXA_ production [37]. Both ST23-KL57 genomes carried *bla*_NDM-1_ and displayed high-level resistance to all carbapenem antibiotics tested.

Isolates harboured between 2 and 8 plasmid replicons. The IncL plasmid was detected in all 73 isolates carrying the *bla*_OXA-48_ gene, and the virulence plasmid (repB_KLEB_VIR) was detected in all isolates. Otherwise, IncF (*n*=41) and IncH (*n*=87) replicon types were the most common, but IncX (*n*=3), IncC (*n*=2) and Col (*n*=17) were also detected. A large number of AMR genes were detected in the predicted plasmid contigs, with isolates carrying up to 17 AMR genes associated with plasmid contigs (mean=3.9, sd=3.8). The most commonly detected AMR genes conferred resistance to aminoglycosides (*aac/aph*), *β*-lactams (*bla*_CTX-M-15_*/bla*_OXA-1_), trimethoprim (*dfrA*), fluroquinolones (*qnr*), sulphonamides (*sul*) and tetracyclines (*tetA*) (see Fig. 1).

MDR was only seen in a small number of the isolates (*n*=15, 17%), but resistance to piperacillin/tazobactam (*n*=76, 86%) and tobramycin (*n*=39, 44%) was common (Table S8). The two ST23-KL57 isolates were significantly more resistant with a greater number of resistance genes (21 and 18), and both were resistant to at least 5 classes of antimicrobials (Tables S5 and S8).

### Phylogenetic analysis

The Irish ST23-KL1 genomes were compared with all ST23-KL1 genome sequences available in the Pathogenwatch database (*n*=424). The Pathogenwatch isolates, which were collected between 1980 and 2023, had a variety of resistance and virulence profiles, but all isolates were classified as hv*Kp*, with 362 isolates (85%) having a Kleborate virulence score of 5 (Table S6).

The phylogenetic comparison carried out using TreeCluster identified a total of 88 clusters (see Fig. S1). There was a median distance of 178 SNPs (range 1–651) between the global collection of ST23 genome sequences. The Irish isolates fell into two distinct clusters. The largest cluster consisted of 72 genomes collected between 2019 and 2023 and had a median SNP distance of 31 SNPs (15–64), of which 68 (94%) were isolated from 8 hospitals located in the South or Southeast of Ireland. Twenty (28%) of these were from sites other than the rectum including blood, urine and sputum. These genomes clustered most closely with genomes from India (83 SNPs), Vietnam (120/124 SNPs) and Canada (134 SNPs).

The second cluster consisted of nine genomes collected between 2020 and 2023 with a median SNP distance of 4.5 SNPS (1–33), which were all associated with three hospitals in the East of Ireland over 3 years (2020–2023). These genomes clustered most closely with genomes from India (78 SNPs), Japan (111 SNPs) and the USA (64 SNPs).

**Fig. 2.**
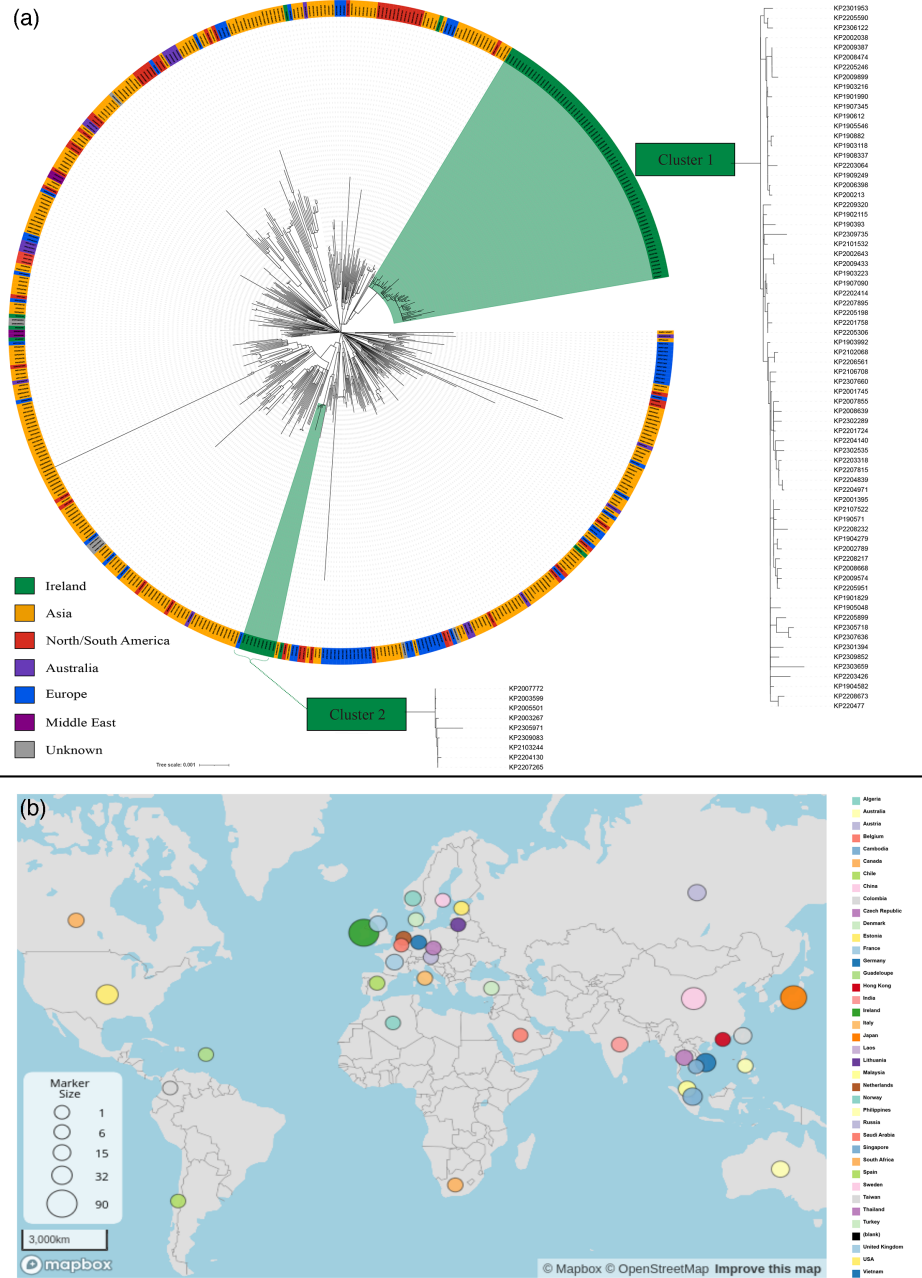
Phylogenetic analysis. **(a)** A core SNP comparison of all ST23-KL1 sequences from Pathogenwatch with the 88 Irish ST23-KL1 *K. pneumoniae* generated using RAxMl-NG with 100 bootstrap replicates. The two main Irish clusters are depicted separately. For bootstrap values and branch lengths, please see Fig. S1 (reference genome: *K. pneumoniae* subsp. *pneumoniae* HS11286). **(b)** A map representing the location of origin of the ST23-KL1 genomes downloaded from the Pathogenwatch database.

The remaining genomes (*n*=7) were found throughout the tree and were often closely related to genomes from various continents (Fig. 2). All but one of these genomes were isolated from an invasive site and were from hospitals not associated with the two main clusters detected. These genomes also differed from the clustered genomes by their lack of carbapenemase genes.

### Hv*Kp* ST23 within patient persistence

Analysis of paired isolates from five different patients revealed the potential for within-patient hv*Kp* persistence and progression from colonization to infection. Five patients had hv*Kp* isolated initially in a rectal swab/faecal sample, thereby reflecting gastrointestinal colonization and subsequently from a site or potential site of infection, blood (*n*=2), foot (*n*=1) and urine (*n*=2), within the same year. These pairs had a maximum SNP distance of 7. A single patient had a sample initially collected from blood and then a liver aspirate within 2 months, which had an SNP distance of 1.

Greater variability in sequence homology was noted in the isolates collected at least 1 year apart. In two pairs where both samples were collected from rectal/faecal samples, an SNP distance of 0 and 8 was detected. The final two pairs of samples were collected at least 1 year apart but had an SNP distance of 22 and 27, respectively. The first pair was collected from a PEG tube site and subsequently from faeces, whilst the second pair was collected from a rectal swab and faeces. These two isolates also displayed genotypic differences in their plasmid and resistance gene content unlike the more closely related isolates (Table S9).

### Virulence of selected isolates in a sepsis model

Eleven isolates were chosen to represent the different clusters detected, sampling times and isolation sources (Table 1). Of the 11 isolates tested in the sepsis model, 9 had an LD_50_ of 1×10^1^–4.1×10^2^ c.f.u., indicating a fully virulent hv*Kp* phenotype. There was no difference in virulence phenotype noted between isolates from the two clusters, nor in isolates that did not form part of any cluster. Colonization isolates from rectal swabs were similarly virulent to those isolated from normally sterile sites, as was an environmental isolate from a shower drain. Two isolates were less virulent with LD_50_s of 1.4×10^4^ and 3.6×10^5^ c.f.u., but these LD_50_s are still consistent with the hv*Kp* pathotype [22]. Of note, these isolates differed from the others tested with respect to the hv*Kp* phenotype-associated *rmpA* gene, which was truncated due to a frameshift mutation caused by a deletion in a poly(G) region of the gene.

**Table 1.** Mortality rates of CD1 mice following subcutaneous challenge with 10^3^ c.f.u. hv*Kp* ST23

Isolate	Source	Location	Cluster	LD50	Kleborate virulence score	Salmochelin locus	Yersiniabactin locus	Aerobactin locus	Colibactin locus	*rmpADC* locus	*rmpA2*	*Peg-344*
KP1901990	Rectal	SouthE1	1	2.30E+02	5	*iro 1*	*ybt 1*	*iuc 1*	*clb 2*	*rmp1*	*rmpA2*_6*−55%	Detected
KP1903118	Blood	SouthE1	1	3.10E+02	5	*iro 1*	*ybt 1*	*iuc 1*	*clb 2*	*rmp1*	*rmpA2*_6–60%	Detected
KP2002038	Blood	SouthE1	1	1.00E+01	5	*iro 1*	*ybt 1*	*iuc 1*	*clb 2*	*rmp1*	*rmpA2*_6*−55%	Detected
KP2002643	Liver abscess	SouthE1	1	4.10E+02	5	*iro 1*	*ybt 1*	*iuc 1*	*clb 2*	*rmp1*	*rmpA2*_6–60%	Detected
KP2003267	Rectal	East2	2	3.60E+5	5	*iro 1*	*ybt 1*	*iuc 1*	*clb 2*	rmp1^†*^	*rmpA2*_6*−47%	Detected
KP2005568	Liver abscess	West1	None	1.00E+01	5	*iro 1*	*ybt 1*	*iuc 1*	*clb 2*	*rmp1*	*rmpA2*_6–60%	Detected
KP2104506	Blood	Mid1	None	3.10E+02	5	*iro 1*	*ybt 1*	*iuc 1*	*clb 2*	*rmp1*	*rmpA2*_3–47%	Detected
KP2204130	Rectal	East2	2	1.00E+01	5	*iro 1*	*ybt 1*	*iuc 1*	*clb 2*	*rmp1*	*rmpA2*_6*−47%	Detected
KP2205198	Shower drain	SouthE1	1	1.00E+01	5	*iro 1*	*ybt 1*	*iuc 1*	*clb 2*	*rmp1*	*rmpA2*_6*−55%	Detected
KP2206007	Vitreous fluid	East9	None	1.00E+01	5	*iro 1*	*ybt 1*	*iuc 1*	*clb 2*	*rmp1*	*rmpA2*_6*−55%	Detected
KP2306122	Urine	West2	1	1.40E+04	5	*iro 1*	*ybt 1*	*iuc 1*	*clb 2*	*rmp1*†	*rmpA2*_6*−55%	Detected

†*rmpA* gene truncated.

## Discussion

The first putative hv*Kp* ST23 isolate in Ireland was detected in 2019. Data presented in this report unequivocally establish that the 11 representative isolates assessed possess the hv*Kp* pathotype as determined by a murine systemic infection model. The genomes of the first detected isolates formed part of a monophyletic cluster in the South East of the country associated with multiple healthcare facilities [38]. In this study, we demonstrate that 80% of the Irish hv*Kp* ST23 detected to date are very closely related (<65 SNP distance) to this original cluster. Our analysis supports the dissemination of this cluster in healthcare facilities primarily in the South and South East, with sporadic occurrence in other regions. In some instances, cases detected in other hospitals have a record or previous association with a healthcare facility in the South East.

A secondary cluster was detected representing 10% of the Irish isolates but was associated with three different healthcare facilities in the East. This is likely to represent a separate introduction and spread of a related but distinct clone in hospitals in the East. Unlike the isolates in the larger cluster, these were not associated with invasive infection, but mainly asymptomatic carriage. The isolates in this cluster also demonstrated almost identical resistance gene, plasmid and antimicrobial susceptibility profiles within the cluster.

The remaining isolates which did not belong to either cluster were from six different healthcare facilities all over the country and generally did not carry carbapenemase genes. This may explain why no non-invasive isolates were detected in these hospitals, since only carbapenemase-positive isolates are routinely sent to the reference laboratory. This could also indicate that ST23 is much more widespread in Irish hospitals and the community than the numbers currently detected as non-CPE isolates are not investigated and hv*Kp* isolates are not routinely screened for.

The analysis also demonstrated that the Irish isolates were linked to diverse geographical regions such as Asia, America and Europe. The main cluster of Irish isolates was most closely related to isolates from India, Canada and Vietnam, whilst the second cluster was closely related to isolates from India, Japan and the USA. This supports previous findings of the dissemination of an ST23 clone globally [18].

Two isolates from a different sublineage were also detected (ST23-KL57), which carried a *bla*_NDM-1_ gene. This is the first incidence of this sublineage detected in Ireland. A recent rapid risk assessment by the ECDC demonstrated that these isolates are likely associated with travel or hospitalization in either Ukraine or Russia [6].

Of concern is the acquisition of carbapenemase genes, which could result in highly virulent organisms which are very difficult to treat, such as the aforementioned ST23-KL57 isolates. These hv isolates carried *bla*_NDM-1_ in combination with at least 14 other AMR genes and were phenotypically resistant to all but 3 antimicrobials. Overall, a small proportion of the Irish ST23 isolates (17%) were MDR, but they mostly retained susceptibility in *in vitro* susceptibility tests to the carbapenems meropenem and imipenem. Most were susceptible to the most commonly used antimicrobial agents. A large proportion of isolates (30%) were from sites of infection. Studies indicate that higher rates of mortality are associated with hv*Kp* infections in the healthcare setting, especially in combination with carbapenemase production [6].

The 11 isolates assessed in the murine systemic infection model demonstrated that infection site and colonization site isolates were true hv*Kp* strains. Similarly, the paired patient samples demonstrated that asymptomatic carriage is possible over long periods of time, with multiple patients demonstrating asymptomatic carriage over a 2-year period. This is of concern in the context of the increased numbers of asymptomatic carriage detected in this study (65%). These asymptomatic carriers serve as a reservoir for infection for both them and others via faecal–oral transmission [39]. These data also support the concept that colonization is often a first step in pathogenesis with the potential for subsequent infection.

The majority of the Irish hv*Kp* ST23-KL1 genomes (86%) carry all five of the virulence genes (*iucA*, *iroB*, *rmpA*, *rmpA2* and *peg-344*) that define a strain as hv*Kp* [22] and possessed the three loci used in the Kleborate classification scheme [19]. Additional virulence genes detected such as type 1 (*fimH*) and type 3 (*mrk*) fimbriae could play a crucial role in both the binding of infecting bacteria to surface molecules on the host cells and in biofilm formation, which may facilitate their survival on surfaces in the hospital environment [40,41]. Importantly, the single environmental isolate assessed in this study (detected in a shower drain from a patient room) possessed the hv*Kp* pathotype as determined by a murine systemic infection model displayed. This raises the concern that acquisition of hv*Kp* could occur via fomites in the healthcare setting and has infection control implications.

Most of the isolates (73%) tested in the mouse systemic infection model displayed a high level of virulence; however, the two isolates were less virulent, albeit their LD_50_s were still consistent with the hv*Kp* pathotype. Interestingly, these isolates were found to possess truncated *rmpA* genes, which are key genes associated with hypervirulence [2,42, 43]

This study has limitations. First, the isolate collection is biassed towards CPE-producing isolates. The development and application of practical low-cost methods to test specifically for rectal colonization with hv*Kp* would be likely to provide a more complete understanding of the dissemination of hv*Kp*; however, such methods are not currently available. Therefore, as this collection includes colonization isolates, it is more representative than most hv*Kp* collections reported to date. Second, the analysis emphasizes those genetic markers of virulence reflected in the established Kleborate score, which is widely used. However, it appears that the pattern of markers *iucA*, *iroB*, *rmpA*, *rmpA2* and *peg-344* correlates more closely with the virulence phenotype in the mouse model [22]. As outlined, this set of markers was present in most hv*Kp* in this collection. Third, the plasmid analysis carried out by Platon is based on short-read assemblies with inherent limitations in plasmid characterization. To fully understand the plasmids present, the resistance genes they carry would require long-read sequencing for full characterization. This could be particularly important in the context of studying the convergence of hv and resistance. Finally, the lack of access to complete patient metadata hampers a full epidemiological investigation. Patient metadata followed data minimization principles to protect patient identity in line with General Data Protection Regulation (GDPR) guidelines.

The detection of these hv*Kp* isolates was mainly incidental and due to their carriage of carbapenemase genes. There is currently no reliable screening protocol for the detection of hv*Kp* in the community or healthcare setting. Preventing the spread of hv*Kp* has proved extremely challenging. Notwithstanding considerable efforts in hospital and hospital networks concerned, cases of colonization and infection with the established clonal group of hv*KP* continue to occur. The absence of reliable and practical methods to detect non-CPE hv*Kp* is a factor that complicates the detection and control of hv*Kp* in the healthcare setting.

## Supplementary material

10.1099/mgen.0.001373 Fig. S1.

10.1099/mgen.0.001373 Uncited Supplementary Material 1.

## Abbreviations

AMR: antimicrobial resistance
CEG: carbapenemase-encoding gene
c*Kp*: classical *Klebsiella pneumoniae*
CPE: carbapenemase-producing Enterobacterales
GRLS: Galway Reference Laboratory Services
hv: hypervirulent
hv*Kp*: hypervirulent *Klebsiella pneumoniae*
MDR: multidrug resistant
MIC: minimum inhibitory concentration
ST23: sequence type 23
