# Supplementary material for: Genomic and phylogenetic analysis of hypervirulent Klebsiella pneumoniae ST23 in Ireland
Source: Microb Genom. 2025 Mar 19;11(3):001373. doi: 10.1099/mgen.0.001373 (PMC12452180; doi:10.1099/mgen.0.001373)

Figure S1: Phylogenetic tree of all K. pneumoniae ST23-KL1 genomes from Irelandand the pathogenwatch database . Branches are labelled with the branch length on top and the bootstrap support value under each branch. Irish isolates are highlited in green.Carbapenemase genes detected are highlited to the right of the genome name. Clusters of genomes are indicated by the different blocks of colour, only clusters with atleast 5 genomes are displayed.

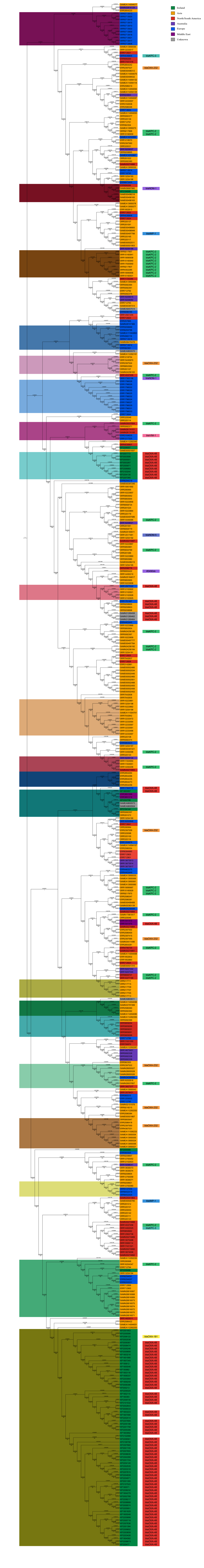

Supplement: Fig. S1. [file mgen-11-01373-s001.pdf]
